# Supplementary material for: Hormonal contraception alters vaginal microbiota and cytokines in South African adolescents in a randomized trial
Source: Nat Commun. 2020 Nov 4;11:5578. doi: 10.1038/s41467-020-19382-9 (PMC7643181; doi:10.1038/s41467-020-19382-9)
Supplement: Supplementary file 1 — Supplementary Information [file 41467_2020_19382_MOESM1_ESM.pdf]

Supplementary Information

Hormonal contraception alters vaginal microbiota and cytokines in South African adolescents in a randomized trial

Balle et al. (2020)

Supplementary Table 1. Transitions between CSTs between baseline and follow up by study arm.

COC arm	CST-I	CST-III	CST-IV	P-value
CST-I	3 (43%)	3 (43%)	1 (14%)	0.504
CST-III	2 (17%)	8 (67%)	2 (17%)	
CST-IV	2 (12%)	6 (35%)	9 (53%)	
**Net-En arm**	**CST-I**	**CST-III**	**CST-IV**	**P-value**
CST-I	7 (100%)	0 (0%)	0 (0%)	0.067
CST-III	4 (57%)	0 (0%)	3 (43%)	
CST-IV	1 (6%)	4 (22%)	13 (72%)	
**CCVR arm**	**CST-I**	**CST-III**	**CST-IV**	**P-value**
CST-I	5 (50%)	2 (20%)	3 (30%)	0.756
CST-III	1 (11%)	5 (56%)	3 (33%)	
CST-IV	1 (7%)	4 (27%)	10 (67%)	

The baseline CST is shown in the first column and the number of transitions to the subsequent groups is shown for each of the CST groups. P values calculated using an omnibus symmetry exact test for a paired contingency table.

Supplementary Table 2. Testing for potential mediators.

**A) sexual risk behavior (condom use).**				
	**Estimate**	**95% CI lower**	**95% CI higher**	**P-value**
**COC vs CCVR (n=66)**				
ACME	0.0127	-0.0832	0.13	0.744
ADE	0.5043	0.1726	0.84	**0.002**
Total Effect	0.5170	0.1714	0.86	**0.010**
Prop. Mediated	0.0164	-0.2149	0.27	0.740
**COC vs Net-En (n=62)**				
ACME	0.0232	-0.0548	0.14	0.590
ADE	0.6010	0.2330	0.96	**<2e-16**
Total Effect	0.6242	02378	0.99	**<2e-16**
Prop. Mediated	0.0217	-0.1384	0.24	0.590
**Net-En vs CCVR (n=60)**				
ACME	-0.0268	-0.1602	0.06	0.58
ADE	-0.0432	-0.4974	0.40	0.86
Total Effect	-0.0700	-0.5510	0.38	0.78
Prop. Mediated	0.0300	-2.8626	2.39	0.80
**B. Time since last menstrual period.**				
	**Estimate**	**95% CI lower**	**95% CI higher**	**P-value**
**COC vs CCVR (n=56)**				
ACME	0.0006	-0.0773	0.09	0.994
ADE	0.4378	0.0618	0.81	**0.028**
Total Effect	0.4385	0.0500	0.81	**0.032**
Prop. Mediated	0.0004	-0.3551	0.28	0.982
**COC vs Net-En (n=53)**				
ACME	0.0021	-0.1009	0.10	0.960
ADE	0.3576	0.0076	0.73	**0.038**
Total Effect	0.3597	-0.0064	0.75	0.054
Prop. Mediated	0.0038	-0.5656	0.51	0.938
**Net-En vs CCVR (n=45)**				
ACME	-0.0125	-0.1819	0.14	0.850
ADE	0.0769	-0.3862	0.56	0.780
Total Effect	0.0644	-0.4395	0.57	0.830
Prop. Mediated	0.0371	-2.8334	4.76	0.850

Mediation analysis was conducted using the R package 'mediation' comparing two arms at the time. ACME, average causal mediation effects; ADE, average direct effects CCVR, combined contraceptive vaginal ring; CI, confidence interval; COC, combined oral contraceptives.

Supplementary Table 3. Characteristics of participants at crossover and exit with microbiome data according to hormonal contraceptive method (per protocol)

	**COC** (n=46)	**Net-En** (n=73)	**CCVR** (n=74)	**P value** COC vs Net-En	**P value** COC vs CCVR	**P value** Net-En vs CCVR
**CSTs**				0.430	0.438	0.900
CST-I	12 (26.1%)	20 (27.4%)	16 (21.6%)			
CST-II	18 (39.1%)	20 (27.4%)	26 (35.1%)			
CSTI-III	16 (34.8%)	33 (45.2%)	32 (43.2%)			
**Vaginal pH, mean (sd)**[1]	4.5 (4.1-5.0)	4.8 (4.2-5.4)	4.9 (4.3-5.4)	**0.010**	**<0.001**	0.193
**Shannon Index, median (IQR)**	0.86 (0.29-1.56)	1.57 (0.72-2.07)	1.60 (0.65-2.06)	**0.004**	**0.002**	0.980
**BV**				0.773	0.927	0.776
BV positive	16 (34.8%)	35 (47.9%)	30 (40.5%)			
BV intermediate	3 (6.52%)	1 (1.37%)	2 (2.70%)			
BV negative	27 (58.8%)	37 (50.7%)	42 (56.8%)			
**HSV-2 serology**	15 (32.6%)	26 (35.6%)	28 (37.8%)	0.910	0.537	0.495
**Yeast cells present**	4 (8.70%)	12 (16.4%)	20 (27.0%)	0.297	**0.035**	0.146
**STI prevalence**						
Any STI(s)	12 (26.1%)	16 (21.9%)	28 (37.8%)	0.491	0.176	**0.026**
Ct	7 (15.2%)	9 (12.3%)	17 (23.0%)	0.673	0.309	0.101
Ng	4 (8.70%)	4 (5.48%)	8 (10.8%)	0.182	0.122	**0.029**
Tv	2 (4.35%)	2 (2.74%)	2 (2.70%)	0.768	0.593	0.760
Mg	1 (2.17%)	3 (4.11%)	2 (2.70%)	0.534	0.728	0.865
**Inflammation category**[2]				0.309	**0.034**	0.136
High	21 (46.7%)	40 (55.6%)	48 (64.9%)			
Low	24 (53.3%)	32 (44.4%)	24 (32.4%)			
**Antibiotic use since last visit**	21 (45.7%)	28 (38.4%)	24 (32.4%)	0.406	0.143	0.438
**Days since last menstrual period, median (IQR)**[3]	19 (10-51)	37 (13-113)	19 (9-113)	0.989	0.971	0.930
**Sexual risk behaviour since last visit**[4]						
Condom use during last PV intercourse	29 (65.9%)	38 (56.7%)	46 (63.9%)	0.199	0.269	0.759
Yes						
Condom use since last visit				0.305	0.595	0.508
Never	5 (11.4%)	23 (34.3%)	21 (29.2%)			
Less than half the time	7 (15.9%)	4 (5.97%)	14 (19.4%)			
Half the time	18 (40.9%)	22 (32.8%)	13 (18.1%)			
More than half the time	4 (9.09%)	9 (13.4%)	7 (9.72%)			
Always	10 (22.7%)	9 (13.4%)	17 (23.6%)			

Binomial mixed effects regression model and conditional logit models of multinomial repsonses were used for the assessment of association of frequency among groups. Linear mixed effects regression models were used for comparison of continous data. BMI, body mass index; BV; bacterial vaginosis; CCVR, combined contraceptive vaginal ring; COC, combined oral contraceptives; CST, community state type; Ct, Chlamydia trachomatis*; E2, oestradiol; FSH, follicle-stimulating hormone, HSV-2, herpes simplex virus type-2 seropositive; IQR, interquartile range; LH, luteinizing hormone; Mg,* Mycoplasma genitalium*; Ng,* Neisseria gonorrhoea*; PV, penile-vaginal; sd, standard deviation; STI, sexually transmitted infection; Tv,* Trichomonas vaginalis*; yrs, years.*

1. Missing data from one visit (one invalid result in the Net-En group)

2. Missing data from four visits (COC, n=1; Net-En, n=1; CCVR, n=2).

3. Missing data from 52 visits (COC, n=3; Net-En, n=26; CCVR, n=23).

4. Missing data from ten visits (COC, n=2; Net-En, n=6; CCVR, n=2).

Supplementary Table 4. Differences in cytokine concentrations A) between baseline and crossover according to study arm and B) between study arms at crossover (intention-to-treat)

A.	COC arm				Net-En arm				CCVR arm			
	Baseline (median (IQR))	Crossover (median (IQR))	P value	BH Adj. P value	Baseline (median (IQR))	Crossover (median (IQR))	P value	BH Adj. P value	Baseline (median (IQR))	Crossover (median (IQR))	P value	BH Adj. P value
**IL-1β**	8.79 (1.81-78.5)	11.0 (0.36-51.3)	0.689	0.960	14.24 (1.82-115)	12.4 (0.58-63.6)	0.074	0.480	1.67 (0.70-55.8)	68.8 (2.88-195)	**0.005**	**0.031**
**IL-6**	0.96 (0.45-2.61)	0.89 (0.36-5.27)	0.876	0.960	1.60 (0.28-9.71)	1.37 (0.21-8.27)	0.775	0.770	0.54 (0.45-3.15)	4.34 (0.90-20.0)	**0.010**	**0.031**
**IL-17A**	1.33 (0.84-2.44)	1.39 (1.02-2.19)	0.961	0.960	1.13 (0.92-4.09)	0.85 (0.59-2.20)	**0.031**	0.410	1.61 (1.01-5.55)	3.67 (1.27-7.97)	0.338	0.340
**IL-17F**	0.34 (0.34-7.18)	0.57 (0.34-10.1)	0.294	0.960	3.82 (0.34-9.40)	0.34 (0.34-9.97)	0.157	0.570	2.11 (0.34-16.4)	7.74 (0.34-27.1)	0.260	0.280
**IL-21**	6.71 (0.68-14.1)	6.86 (2.42-11.2)	0.674	0.960	7.22 (2.49-10.8)	2.87 (0.01-14.2)	0.317	0.570	5.24 (2.72-9.66)	12.8 (6.24-24.8)	**0.007**	**0.031**
**IL-22**	6.08 (4.03-10.2)	6.25 (4.40-9.15)	0.606	0.960	6.70 (5.16-11.9)	6.70 (4.02-9.73)	0.507	0.660	6.82 (4.55-14.5)	10.7 (5.10-20.9)	0.084	0.100
**IL-23**	0.83 (0.12-11.1)	0.59 (0.12-9.31)	0.559	0.960	3.56 (0.12-10.5)	3.15 (0.12-9.07)	0.242	0.570	0.68 (0.12-9.05)	6.40 (0.12-14.1)	0.070	0.100
**IL-25**	0.08 (0.01-0.49)	0.01 (0.01-0.40)	0.830	0.960	0.20 (0.01-0.50)	0.01 (0.01-0.57)	0.572	0.680	0.24 (0.01-0.37)	0.39 (0.02-1.41)	0.060	0.098
**IL-31**	41.6 (22.1-61.5)	34.5 (21.0-65.8)	0.919	0.960	34.5 (21.1-49.7)	26.1 (17.1-56.7)	0.350	0.570	35.6 (26.1-616)	60.8 (23.3-104)	0.086	0.100
**IL-33**	4.37 (1.68-8.24)	3.65 (1.13-7.71)	0.695	0.960	4.10 (1.15-10.0)	3.48 (0.23-9.72)	0.236	0.570	3.48 (1.15- 12.1)	9.56 (3.65-21.1)	**0.026**	0.057
**IFN-γ**	1.76 (1.02-4.14)	1.81 (0.95-3.55)	0.891	0.960	2.68 (1.45-4.90)	2.11 (0.49-4.15)	0.313	0.570	2.64 (1.01-3.45)	3.36 (1.46-8.50)	**0.020**	0.052
**sCD40L**	4.57 (0.07-7.51)	2.50 (0.07-8.46)	0.829	0.960	0.47 (0.07-5.57)	0.07 (0.07-7.60)	0.627	0.680	1.41 (0.07-6.77)	7.21 (0.07-12.9)	0.056	0.098
**TNF-α**	0.45 (0.15-2.09)	0.30 (0.14-1.62)	0.330	0.960	1.35 (0.14-5.60)	1.40 (0.05-3.31)	0.462	0.660	0.43 (0.06-2.05)	1.21 (0.32-8.36)	**0.009**	**0.031**

B.	COC vs Net-En			COC vs CCVR			Net-En vs CCVR		
	P value	[1]BH Adj P value	[2]LME model p value	P value	[1]BH Adj P value	[2]LME model p value	P value	[1]BH Adj P value	[2]LME model p value
**IL-1β**	0.918	0.920	0.670	**0.016**	0.095	0.437	**0.037**	0.120	0.061
**IL-6**	0.863	0.920	0.938	**0.022**	0.095	0.291	0.058	0.130	0.089
**IL-17A**	0.126	0.230	0.520	**0.048**	0.130	1.000	**0.001**	**0.031**	0.248
**IL-17F**	0.480	0.590	0.520	0.070	0.150	1.000	**0.006**	0.095	0.499
**IL-21**	0.457	0.590	0.520	**0.018**	0.095	0.291	**0.011**	0.095	**0.007**
**IL-22**	0.778	0.870	0.520	0.137	0.230	0.794	0.054	0.130	0.750
**IL-23**	0.923	0.920	0.670	0.103	0.210	1.000	0.163	0.270	0.372
**IL-25**	0.766	0.870	0.679	**0.014**	0.095	0.291	**0.028**	0.100	**0.016**
**IL-31**	0.345	0.480	0.520	0.176	0.280	0.291	0.055	0.130	**0.009**
**IL-33**	0.566	0.670	0.520	**0.022**	0.095	0.291	**0.011**	0.095	**0.007**
**IFN-γ**	0.904	0.920	0.520	0.108	0.210	0.618	0.128	0.230	**0.023**
**sCD40L**	0.424	0.570	0.670	0.190	0.280	0.414	0.057	0.130	**0.041**
**TNF-α**	0.485	0.590	0.931	**0.026**	**0.026**	0.437	0.264	0.380	0.169

Cytokine concentrations in pg/ml. P values calculated usingtwo-tailed paired Wilcoxon-Rank-Sum tests (A) and unpaired Mann-Whitney U tests (B). COC, n=36; Net-En, n=35; CCVR, n=34.

[1]Unpaired Man-Whitney U tests adjusted for multiple comparison using the Benjamini-Hochberg (BH) method.

2Linear Mixed Effects (LME) model adjusted for antibiotic use, baseline alpha diversity and condom use amd adjusted for multiple comparisons using the Benjamini-Hochberg (BH) method.

Supplementary Table 5. Multivariate logistic regression analyses.

ITT crossover	OR	95% CI	P value
**COC vs CCVR**			
High Inflammation	3.1	1.0-10.9	0.072
CST-I vs CST-III	0.5	0.1-1.8	0.437
CST-I vs CST-IV	0.5	0.1-2.4	0.288
**COC vs Net-En**			
High Inflammation	1.6	0.4-6.5	0.488
CST-I vs CST-III	0.1	0.02-0.5	**0.009**
CST-I vs CST-IV	0.5	0.1-2.1	0.343
**Net-En vs CCVR**			
High Inflammation	2.1	0.6-8.3	0.272
CST-I vs CST-III	3.8	0.8-19.5	0.109
CST-I vs CST-IV	1.0	0.2-4.5	0.962

Supplementary Table 6. Primer sequences for 16S rRNA amplicon sequencing of the V4 region

Primer	Primer sequence (5' to 3')
**Primary PCR primers**	
Modified 515F	GTGCCAGCMGCCGCGGTAA
Modified 806R	GGACTACHVGGGTWTCTAAT
**Secondary PCR primers**	
501	AATGATACGGCGACCACCGAGATCTACAC**TAGATCGC**TCGTCGGCAGCGTC
502	AATGATACGGCGACCACCGAGATCTACAC**CTCTCTAT**TCGTCGGCAGCGTC
503	AATGATACGGCGACCACCGAGATCTACAC**TATCCTCT**TCGTCGGCAGCGTC
504	AATGATACGGCGACCACCGAGATCTACAC**AGAGTAGA**TCGTCGGCAGCGTC
505	AATGATACGGCGACCACCGAGATCTACAC**GTAAGGAG**TCGTCGGCAGCGTC
506	AATGATACGGCGACCACCGAGATCTACAC**ACTGCATA**TCGTCGGCAGCGTC
507	AATGATACGGCGACCACCGAGATCTACAC**AAGGAGTA**TCGTCGGCAGCGTC
508	AATGATACGGCGACCACCGAGATCTACAC**CTAAGCCT**TCGTCGGCAGCGTC
701	CAAGCAGAAGACGGCATACGAGAT**TCGCCTTA**GTCTCGTGGGCTCGG
702	CAAGCAGAAGACGGCATACGAGAT**CTAGTACG**GTCTCGTGGGCTCGG
703	CAAGCAGAAGACGGCATACGAGAT**TTCTGCCT**GTCTCGTGGGCTCGG
704	CAAGCAGAAGACGGCATACGAGAT**GCTCAGGA**GTCTCGTGGGCTCGG
705	CAAGCAGAAGACGGCATACGAGAT**AGGAGTCC**GTCTCGTGGGCTCGG
706	CAAGCAGAAGACGGCATACGAGAT**CATGCCTA**GTCTCGTGGGCTCGG
707	CAAGCAGAAGACGGCATACGAGAT**GTAGAGAG**GTCTCGTGGGCTCGG
708	CAAGCAGAAGACGGCATACGAGAT**CCTCTCTG**GTCTCGTGGGCTCGG
709	CAAGCAGAAGACGGCATACGAGAT**AGCGTAGC**GTCTCGTGGGCTCGG
710	CAAGCAGAAGACGGCATACGAGAT**CAGCCTCG**GTCTCGTGGGCTCGG
711	CAAGCAGAAGACGGCATACGAGAT**TGCCTCTT**GTCTCGTGGGCTCGG
712	CAAGCAGAAGACGGCATACGAGAT**TCCTCTAC**GTCTCGTGGGCTCGG

The adapter sequences are underlined in the 501 and 701 secondary primer sequences. The same sequences are found in each of the 500 and 700 primer series. The 8-nucleotide sample indices are bolded.

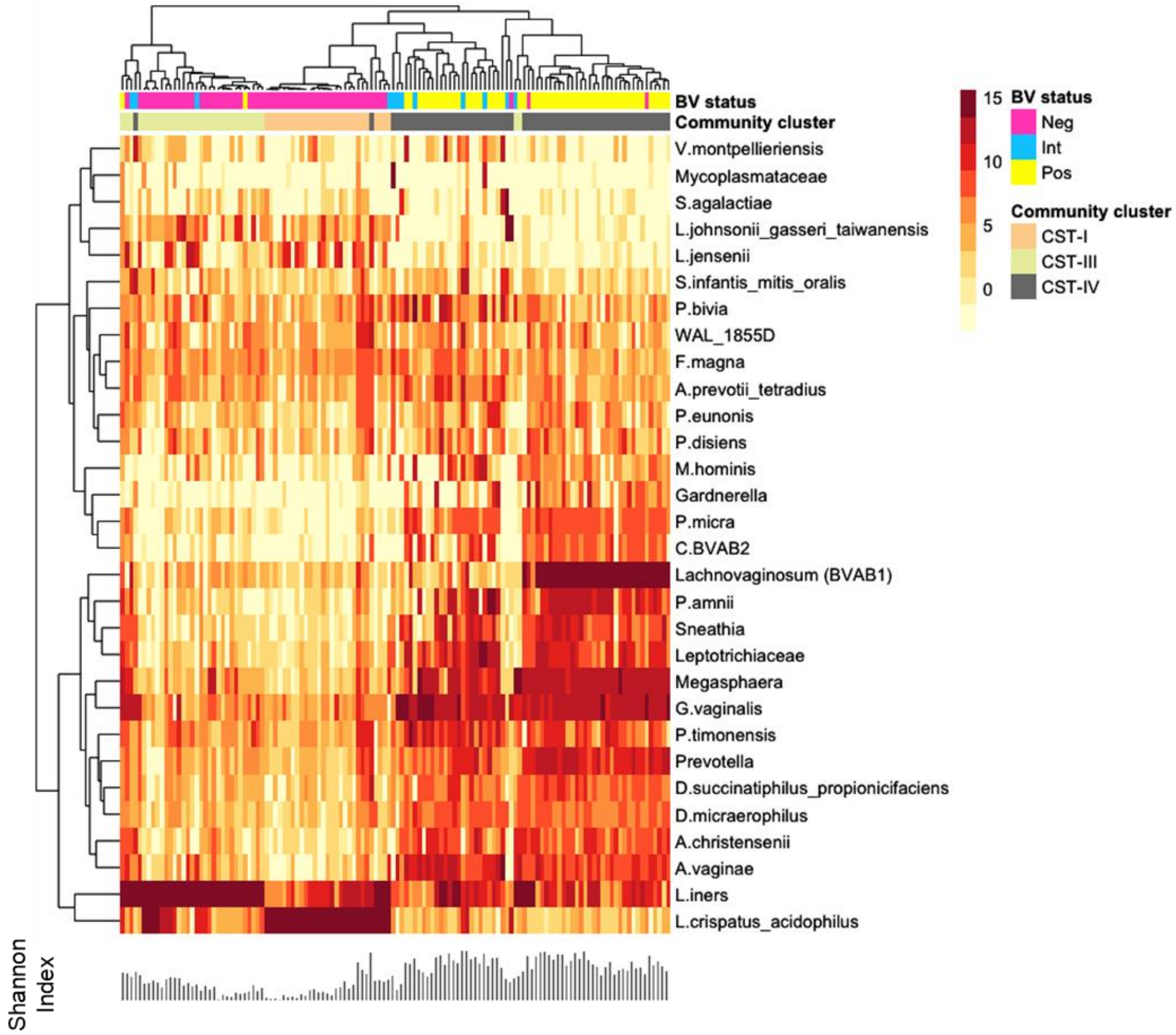

Supplementary Figure 1. Composition of vaginal lateral wall microbiota at baseline. Heatmap of the 30 most abundant taxa (rows) identified by 16S rRNA microbiome profiling using unsupervised hierarchical clustering with weighted UniFrac distances in all samples (columns). Unsupervised hierarchical clustering of vaginal lateral wall samples from 126 participants. The dendrogram was generated using average linkage clustering with weighted UniFrac distance, based on the relative abundance of taxa (merged at lowest taxonomic level) in each sample. Log2-transformed standardized read counts are illustrated by the colour key. Annotation bars above the heatmap depict BV status based on Nugent scoring (upper bar) and community state type (bottom bar). Source data are provided as a Source Data file.

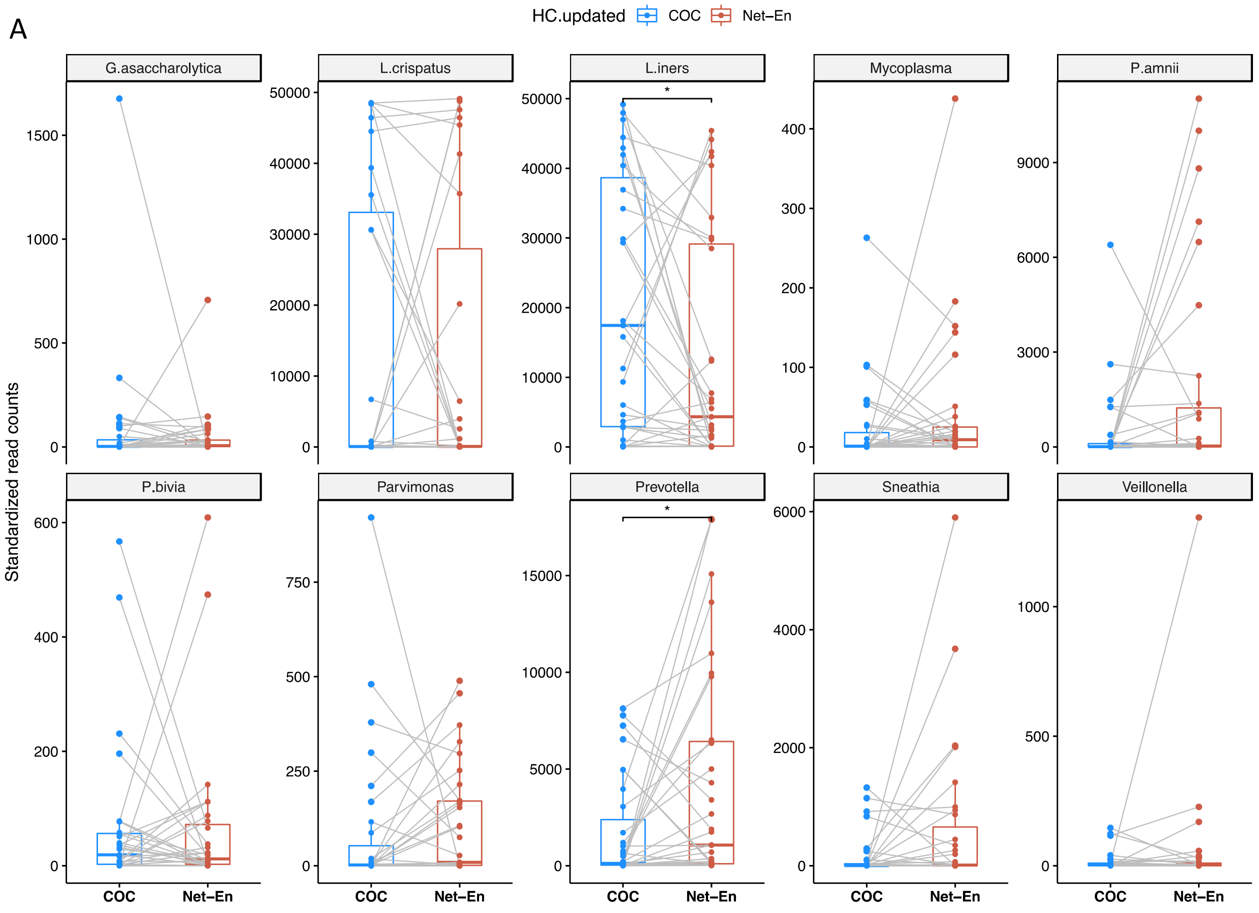
A
HC.updated COC Net−En
G.asaccharolytica
L.crispatus
L.iners
Mycoplasma
P.amnii
P.bivia
Parvimonas
Prevotella
Sneathia
Veillonella
Standardized read counts
COC
Net−En

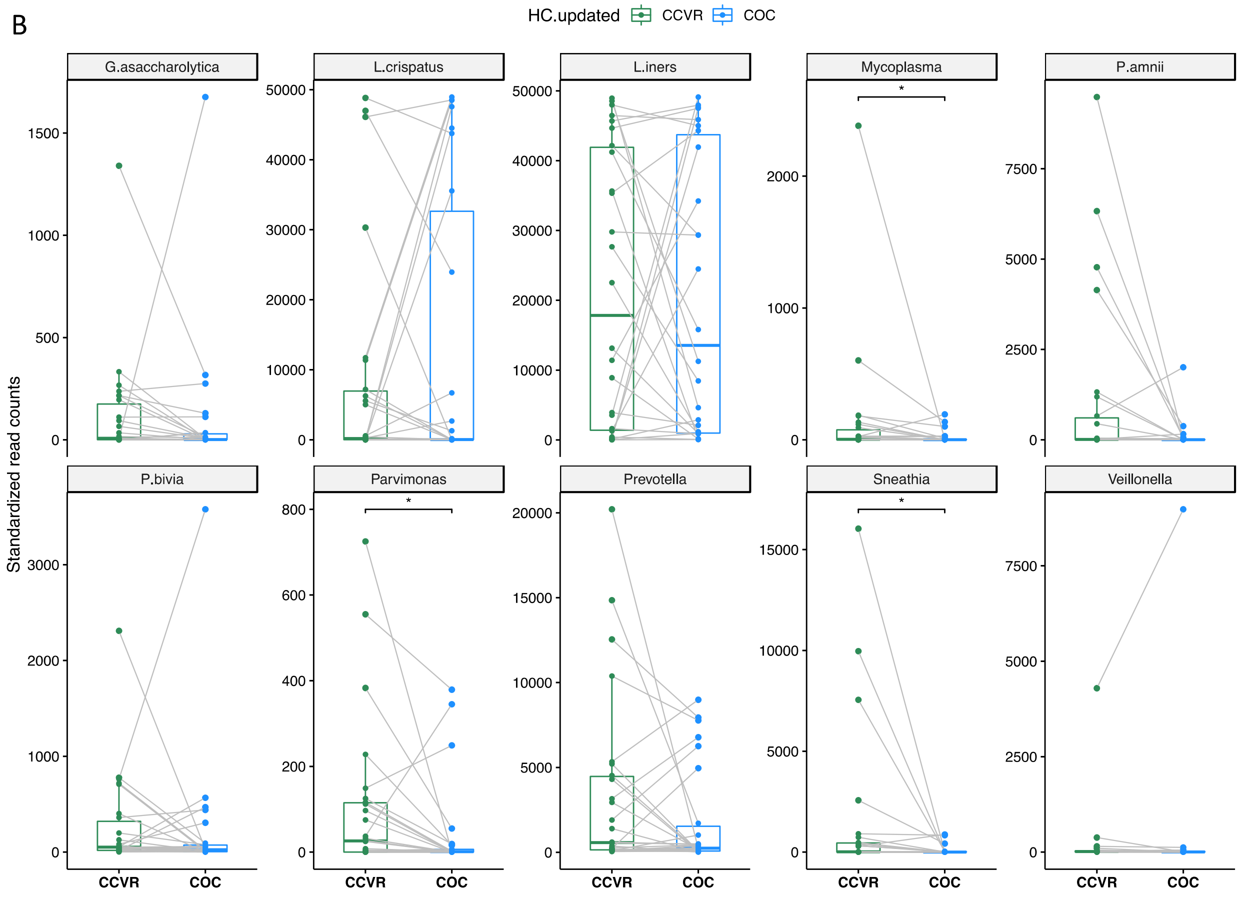
B
HC.updated CCVR COC
G.asaccharolytica
L.crispatus
L.iners
Mycoplasma
P.amnii
P.bivia
Parvimonas
Prevotella
Sneathia
Veillonella
Standardized read counts
CCVR
COC

C

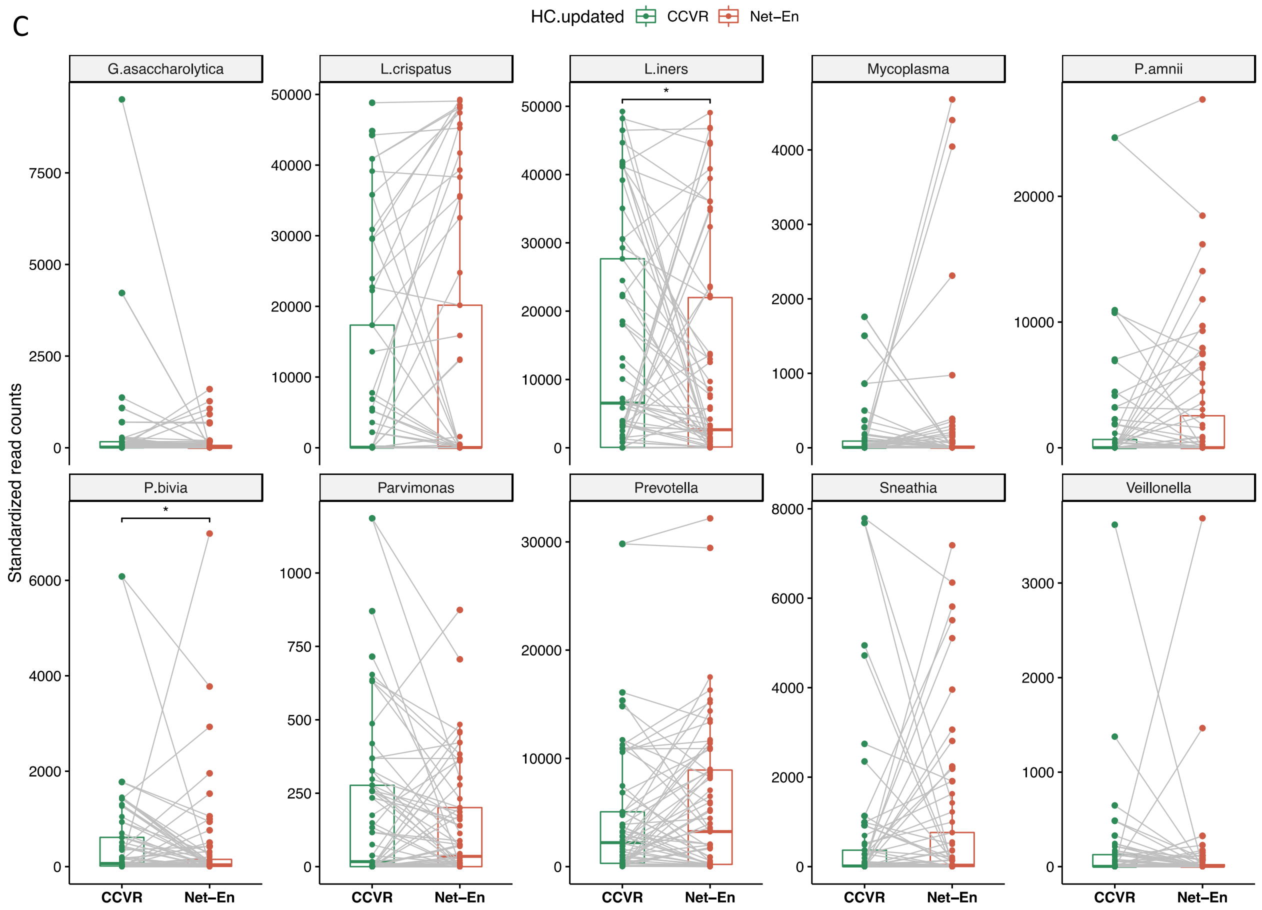

Supplementary Figure 2. Differences in standardized read counts of bacteria of interest within participants changing between specific contraceptive methods. Boxplots showing the standardized read counts of bacteria of interest in A) 62 vaginal samples from 29 participants changing from COC to Net-En or vice versa (p=0.031, p=0.032, respectively, for those denoted with bar), B) 52 vaginal samples from 26 participants changing from COC to CCVR or vice versa (p=0.019, p=0.017, p=0.020, respectively, for those denoted with bar) and C) 130 vaginal samples from 50 participants changing from Net-En to CCVR or vice versa (p=0.045, p=0.046, respectively, for those denoted with bar). P values were generated using two-tailed paired Wilcoxon Signed-Rank tests adjusted for multiple comparisons using the Benjamini-Hochberg (BH) method. Significance codes: *: p<0.05. CCVR, combined contraceptive vaginal ring; COC, combined oral contraceptives. The lower and upper hinges of the boxplots correspond to the first and third quartiles (25th and 75th percentiles), the middle lines of the boxes indicate median, top and bottom whiskers demonstrate largest and lowest value no further than 1.5*interquatiles range from the hinge. Data beyond the end of the whiskers are plotted individually. Source data are provided as a Source Data file.

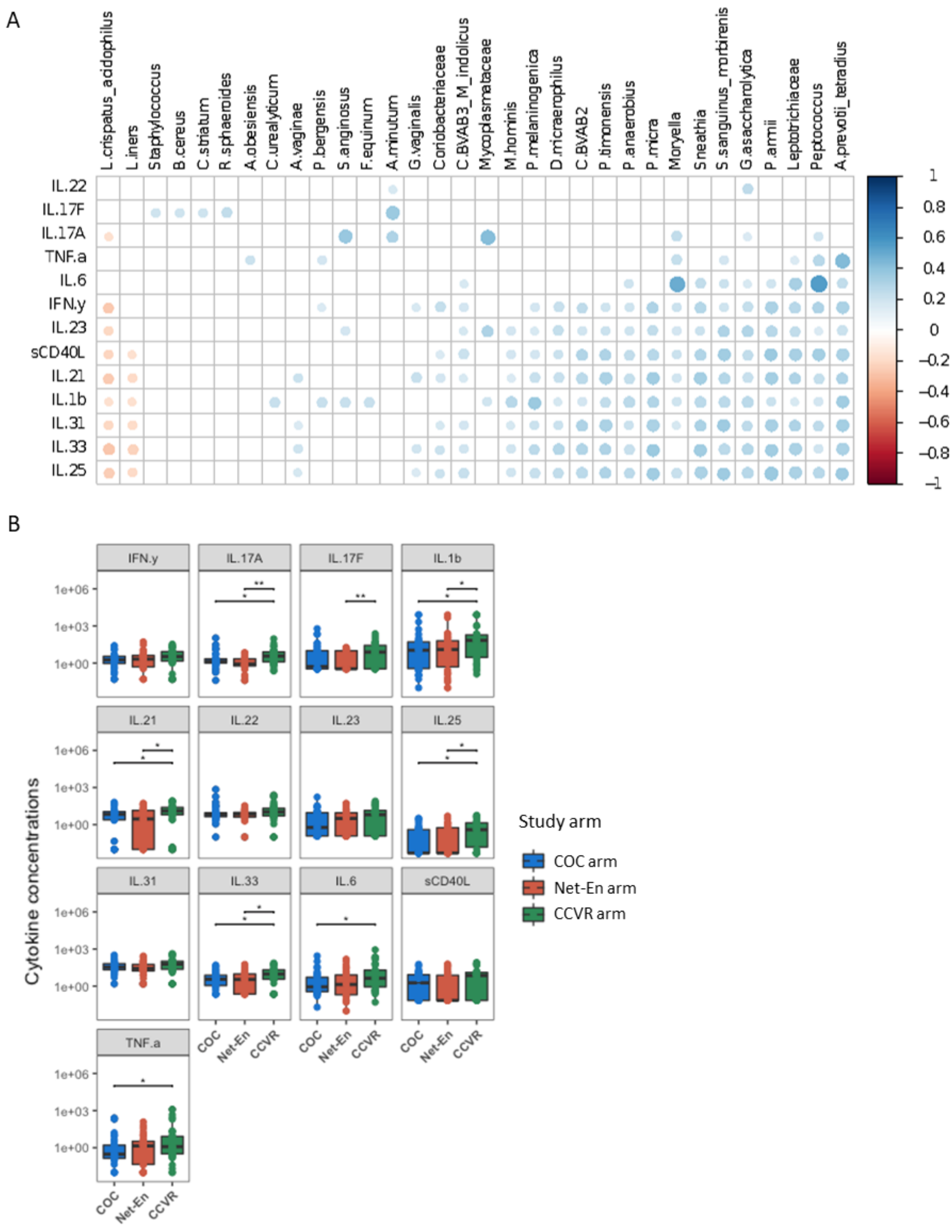

Supplementary Figure 3. Association of bacterial taxa, hormonal contraception and cytokine levels. A) Correlation between cytokines and standardized read counts of individual bacteria. Significant positive (blue) or negative (red) correlations between standardized read counts of bacteria (merged at lowest taxonomic level) and vaginal concentrations of cytokines using Spearman's rank correlation. Adjusted p value of 0.1 set as cut-off. Only R^2 values>0.2 displayed. Each OTU has ≥10 counts in ≥30% of samples. **B**) Cytokine concentrations according to study arm at crossover in an intention-to-treat analysis (COC, n=36; Net-En, n=35; CCVR, n=34). Concentrations in pg/ml. P values were calculated using two-tailed Mann-Whitney U tests adjusted for multiple comparisons using the Benjamini-Hochberg (BH) method. (p=0.048, p=0.001, p=0.006, p=0.016, p=0.037, p=0.018, p=0.011, p=0.014, p=0.028, p=0.022, p=0.011, p=0.022, p=0.026, respectively, for those denoted with bar). Significance codes: *: p<0.05, **: p<0.01, ***: p<0.001. The lower and upper hinges of the boxplots correspond to the first and third quartiles (25th and 75th percentiles), the middle lines of the boxes indicate median, top and bottom whiskers demonstrate largest and lowest value no further than 1.5 * interquartile range from the hinge. Data beyond the end of the whiskers are plotted individually. CCVR, combined contraceptive vaginal ring; COC, combined oral contraceptives. Source data are provided as a Source Data file.

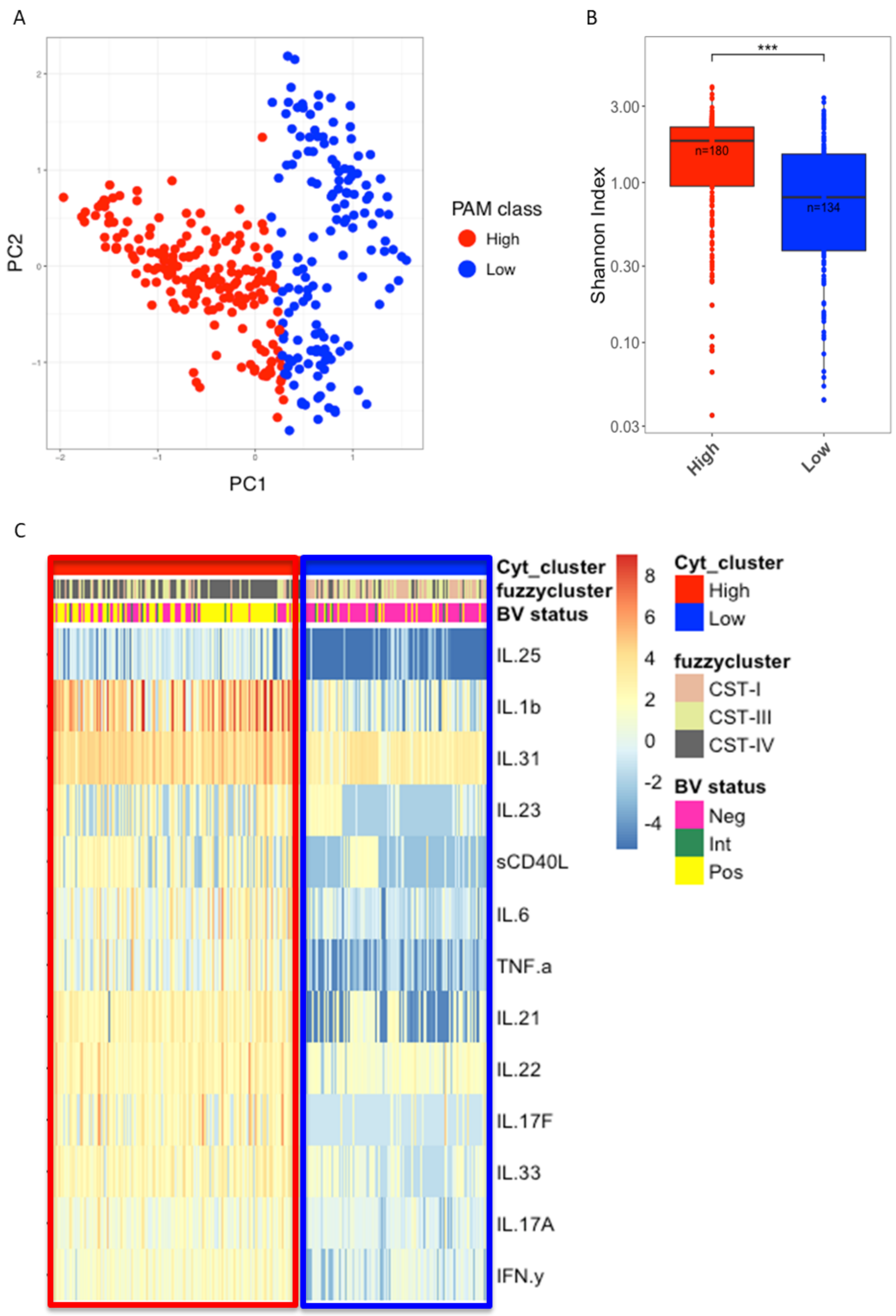

Supplementary Figure 4. Inflammation clusters. A) PCoA of the measured cytokines (n=323 from 130 adolescents) coloured by inflammation group (High, n=180 (red); Low, n=143 (blue)) generated by partitioning around medoids (PAM) clustering with Euclidean distances. **B**) Boxplot showing the alpha diversity measured using Shannon Index of the microbiota from 323 vaginal samples from 130 adolescents according to inflammation group (High, n=180 (red); Low, n=143 (blue)) identified by PAM clustering using Euclidean distances of the cytokine data. Y-axis log10-transformed. P values were calculated using two-tailed Mann-Whitney U test (p=6.253e-12). ***=p<0.001. CCVR, combined contraceptive vaginal ring; COC, combined oral contraceptives. **C**) Heatmap of cytokine concentrations of thirteen cytokines from a Th17 bioplex panel from 323 samples from 130 adolescents using average linkage clustering with Euclidean distances. Annotation bars above the heatmap depict inflammation group generated by PAM clustering using Euclidean distances of the cytokine data (Cyt_cluster, top bar), community cluster identified using soft k-means clustering using weighted UniFrac distances (fuzzycluster, middle bar), BV status based on Nugent scoring (lower bar). Log2-transformed cytokine concentrations are illustrated by the colour key.
