# Supplementary material for: Hormonal contraception alters vaginal microbiota and cytokines in South African adolescents in a randomized trial
Source: Nat Commun. 2020 Nov 4;11:5578. doi: 10.1038/s41467-020-19382-9 (PMC7643181; doi:10.1038/s41467-020-19382-9)
Supplement: Supplementary file 3 — Reporting Summary [file 41467_2020_19382_MOESM3_ESM.pdf]

nature research

Corresponding author(s): Heather B. Jaspan

Last updated by author(s): Sep 29, 2020

Reporting Summary

Nature Research wishes to improve the reproducibility of the work that we publish. This form provides structure for consistency and transparency in reporting. For further information on Nature Research policies, see Authors & Referees and the Editorial Policy Checklist .

Statistics

For all statistical analyses, confirm that the following items are present in the figure legend, table legend, main text, or Methods section.

n/a	Confirmed	
☐	☒	The exact sample size (*n*) for each experimental group/condition, given as a discrete number and unit of measurement
☒	☐	A statement on whether measurements were taken from distinct samples or whether the same sample was measured repeatedly
☐	☒	The statistical test(s) used AND whether they are one- or two-sided<br>*Only common tests should be described solely by name; describe more complex techniques in the Methods section.*
☐	☒	A description of all covariates tested
☐	☒	A description of any assumptions or corrections, such as tests of normality and adjustment for multiple comparisons
☐	☒	A full description of the statistical parameters including central tendency (e.g. means) or other basic estimates (e.g. regression coefficient) AND variation (e.g. standard deviation) or associated estimates of uncertainty (e.g. confidence intervals)
☐	☒	For null hypothesis testing, the test statistic (e.g. *F*, *t*, *r*) with confidence intervals, effect sizes, degrees of freedom and *P* value noted<br>*Give P values as exact values whenever suitable.*
☒	☐	For Bayesian analysis, information on the choice of priors and Markov chain Monte Carlo settings
☐	☒	For hierarchical and complex designs, identification of the appropriate level for tests and full reporting of outcomes
☐	☒	Estimates of effect sizes (e.g. Cohen's *d*, Pearson's *r*), indicating how they were calculated

Our web collection on statistics for biologists contains articles on many of the points above.

Software and code

Policy information about availability of computer code

Data collection	No software used
Data analysis	Data analysis was conducted using usearch7, UCHIME, RDP classifier, UPARSE and QIIME 1.8.0 using an in-house pipeline (https://github.com/uct-cbio/16S-rDNA-pipeline), NCBI's BLAST tool, Bio-plex manager software version 4 and custom scripts in R version 3.5.3 (http://github.com/frk.balle/uCHOOSE).

For manuscripts utilizing custom algorithms or software that are central to the research but not yet described in published literature, software must be made available to editors/reviewers. We strongly encourage code deposition in a community repository (e.g. GitHub). See the Nature Research guidelines for submitting code & software for further information.

Data

Policy information about availability of data

All manuscripts must include a data availability statement. This statement should provide the following information, where applicable:

- Accession codes, unique identifiers, or web links for publicly available datasets
- A list of figures that have associated raw data
- A description of any restrictions on data availability

Raw sequence data for 16S rRNA gene amplicon sequences are available at http://www.ebi.ac.uk/ under project number PRJEB30774. De-identfied participant metadata and cytokine data is available in the source data file. R analysis scripts available on http://github.com/frk.balle/uCHOOSE. The parent study protocol can be found at https://clinicaltrials.gov/ct2/show/record/NCT02404038. The source data underlying Figs 2, 3, and 4, Tables 1 and 2, Supplementary Figs 1, 2, 3 and 4 and Supplementary Tables 1, 2, 3, 4 and 5 are provided as a Source Data file.

Field-specific reporting

Please select the one below that is the best fit for your research. If you are not sure, read the appropriate sections before making your selection.

- [x] Life sciences
- [ ] Behavioural & social sciences
- [ ] Ecological, evolutionary & environmental sciences

For a reference copy of the document with all sections, see nature.com/documents/nr-reporting-summary-flat.pdf

Life sciences study design

All studies must disclose on these points even when the disclosure is negative.

Sample size	The sample size of the substudy was limited to that of the parent study, where power calculations were based on the study's primary outcome, the relative acceptability of CCVR versus other modalities based on the total score for the ORTHO BC SAT questionnaire at 4 months after randomization. Power for the substudy was calculated for the aim of evaluating CD4+ cervical T cell populations at 4 months post-contraceptive method initiation as prior data on the microbial endpoints were not available. Based on estimates from previous research, n=50 per group provides 80% power to detect absolute differences of 10% in the mean frequencies of CD4+ HLADR+CD38+ cervical T cells between any two contraceptive groups by one-way ANOVA at alpha=0.05.
Data exclusions	Data from participants not eligible for enrollment in the parent study were excluded.
Replication	The findings cannot be reproduced. This is a highly individual cohort that to our knowledge has never been replicated. Due to limited sample availability, sequencing and luminex analysis was not performed in replicates.
Randomization	Enrolled participants were randomized into three study arms in a 1:1:1 fashion. Randomisation was performed using random number sequence in Stata and provided to the pharmacist in sealed envelopes.
Blinding	This was an open-label randomized trial to assess acceptability and feasibility of various hormonal contraceptives in South African adolescents. Therefore there was no blinding. However, laboratory assays were performed in a blinded fashion.

Reporting for specific materials, systems and methods

We require information from authors about some types of materials, experimental systems and methods used in many studies. Here, indicate whether each material, system or method listed is relevant to your study. If you are not sure if a list item applies to your research, read the appropriate section before selecting a response.

Materials & experimental systems

n/a	Involved in the study	
[x]	[ ]	Antibodies
[x]	[ ]	Eukaryotic cell lines
[x]	[ ]	Palaeontology
[x]	[ ]	Animals and other organisms
[ ]	[x]	Human research participants
[ ]	[x]	Clinical data

Methods

n/a	Involved in the study	
[x]	[ ]	ChIP-seq
[x]	[ ]	Flow cytometry
[x]	[ ]	MRI-based neuroimaging

Human research participants

Policy information about studies involving human research participants

Population characteristics	150 females between ages of and including 15-19 years were approached from within a funded, randomized controlled trial, CHAMPS UChoose. All enrolled in the parent trial were approached to participate in this study. The proposed informal settlement where the research took place was chosen because of its high HIV prevalence (30%) since this research is relevant to adolescents at high risk for HIV.
Recruitment	The adolescents enrolled in this study were recruited through a parent study, UChoose, which is an open-label, randomized crossover study with the purpose of evaluating the feasibility of different hormonal contraception options among adolescents (clinicaltrials.gov/NCT02404038; PI: LG Bekker). Due to the randomized design, we were poised to overcome many of the biases that have plagued prior observational studies, such as confounding due to reductions in condom use by women using more effective contraceptives. Yet, although this study was randomized, it was not blinded, and participants may have altered their sexual risk behavior due to perceived risk for pregnancy with use of different and potentially unfamiliar hormonal contraceptive methods. However, we found that these potential mediators could only explain a very small and non-significant proportion of the effects of study arm on the microbiota. Adherence to randomization arm tended to be poorer for those using the ring, as has been reported in other trials. However, this study differs from many of these prior ring studies as the adolescents in this study were seeking effective contraception and knew they had the risk of pregnancy with non-adherence. Additionally, since an aim of the parent study was to assess feasibility and acceptability of methods in adolescents, reporting of problems with adherence and method changes in response were encouraged, albeit reliant on self-report.
Ethics oversight	Approval for the study was obtained from the Human Research Ethics Committee at the University of Cape Town (HREC 801/2014)

Note that full information on the approval of the study protocol must also be provided in the manuscript.

Clinical data

Policy information about clinical studies

All manuscripts should comply with the ICMJEguidelines for publication of clinical research and a completedCONSORT checklist must be included with all submissions.

Clinical trial registration	clinicaltrials.gov/NCT02404038
Study protocol	https://clinicaltrials.gov/ct2/show/record/NCT02404038
Data collection	Samples were collected from adolescent females attending the Desmond Tutu HIV Foundation (DTHF) Youth Centre in Masiphumelele, Cape Town, South Africa, between July 2015 to June 2017. Masiphumelele is a low income, high-density area with a high HIV prevalence (30%) placing the local youth at high risk of HIV. Recruitment of participants took place from the family planning clinic and other clinics housed within the community centre.
Outcomes	Primary (for parent trial): 13-item Ortho Birth Control satisfaction assessment tool to measure acceptability of each contraceptive option.<br>Secondary (for parent trial): self-report, returned pill packs and rings to measure adherence to each contraceptive method [Time Frame: 8 weeks, 16 weeks, 24 weeks & 32 weeks] interviewer-administered questionnaire on adherence to product, pill counts and visual inspection of rings self-reported measure of the impact of contraceptive method use on sexual risk behavior [Time Frame: baseline, 8 weeks, 16 weeks, 24 weeks & 32 weeks] interviewer-administered questionnaires measuring sexual risky behavior, ie. number of sexual partners, condom use, alcohol use qualitative measures of perceptions on key issues associated with each method of contraceptive measured in focus group discussions [Time Frame: 8 months]<br>Primary outcomes (substudy): cervical T cell activation, vaginal microbial diversity and cytokine concentrations.
